# Pull-off and friction forces of micropatterned elastomers on soft substrates: the effects of pattern length scale and stiffness

**DOI:** 10.3762/bjnano.10.8

**Published:** 2019-01-08

**Authors:** Peter van Assenbergh, Marike Fokker, Julian Langowski, Jan van Esch, Marleen Kamperman, Dimitra Dodou

**Affiliations:** 1 Biomechanical Engineering Department, Delft University of Technology, Mekelweg 2, 2628 CD Delft, The Netherlands; 2 Experimental Zoology Group, Wageningen University & Research, De Elst 1, 6708 WD Wageningen, The Netherlands; 3 Chemical Engineering Department, Delft University of Technology, Van der Maasweg 9, 2629 HZ Delft, The Netherlands; 4 Polymer Science, Zernike Institute for Advanced Materials, University of Groningen, Nijenborgh 4, 9747 AG Groningen, The Netherlands

**Keywords:** adhesion, biomimetic micropatterned adhesive, colloidal lithography, friction, pull-off, soft substrate

## Abstract

The adhesiveness of biological micropatterned adhesives primarily relies on their geometry (e.g., feature size, architecture) and material properties (e.g., stiffness). Over the last few decades, researchers have been mimicking the geometry and material properties of biological micropatterned adhesives. The performance of these biomimetic micropatterned adhesives is usually tested on hard substrates. Much less is known about the effect of geometry, feature size, and material properties on the performance of micropatterned adhesives when the substrate is deformable. Here, micropatterned adhesives of two stiffness degrees (Young’s moduli of 280 and 580 kPa) were fabricated from poly(dimethylsiloxane) (PDMS) and tested on soft poly(vinyl alcohol) (PVA) substrates of two stiffness degrees (12 and 18 kPa), and on hard glass substrates as a reference. An out-of-the-cleanroom colloidal lithographic approach was successfully expanded to fabricate adhesives with two geometries, namely dimples with and without a terminal layer. Dimples without a terminal layer were fabricated on two length scales, namely with sub-microscale and microscale dimple diameters. The cross section of samples with a terminal layer showed voids with a spherical shape, separated by hourglass-shaped walls. These voids penetrate the terminal layer, resulting in an array of holes at the surface. We found that on soft substrates, generally, the size of the dimples did not affect pull-off forces. The positive effects of sub-microscale features on pull-off and friction forces, such as defect control and crack trapping, as reported in the literature for hard substrates, seem to disappear on soft substrates. The dimple geometry with a terminal layer generated significantly higher pull-off forces compared to other geometries, presumably due to interlocking of the soft substrate into the holes of the terminal layer. Pull-off from soft substrates increased with the substrate stiffness for all tested geometries. Friction forces on soft substrates were the highest for microscale dimples without a terminal layer, likely due to interlocking of the soft substrate between the dimples.

## Introduction

### Pull-off and friction forces of micropatterned adhesives as a function of geometry, feature size, and stiffness

Over the last few decades, researchers have been developing micropatterned adhesives mimicking the geometry and material properties of biological dry adhesives [1–5]. Pull-off and friction forces of these biomimetic adhesives rely on the formation of intimate contact with the substrates [6], enabling physical interactions between the adhesive and the substrate, in the form of intermolecular forces, capillary forces, and suction forces. To achieve intimate contact between the adhesive and the substrate, researchers have been designing micropatterned adhesives with a low effective elastic modulus *E*_eff_ [6]. For example, micro- and/or nanoscale fibrillar geometries have been reported [7], where the flexibility of the individual fibrils leads to a low *E*_eff_ [8]. Furthermore, micropatterns with a fibrillar geometry have been shown to have better defect control [9] and better stress distribution [10] compared to smooth adhesives. The decreased *E*_eff_ of a fibrillar geometry also leads to decreased contact stiffness [11] and higher conformability to substrate roughness [12].

The abovementioned effects of fibrillary geometries can be further enhanced with altering the pillar geometry. For example, Gorb et al. fabricated micropillars of 100 μm height and a stem diameter of 60 μm, terminated with a thin (2 μm) disc of 40 μm in diameter [11]. These so-called mushroom-shaped micropillars generated higher pull-off forces than flat-punch micropillars, a phenomenon attributed to a higher adaptability to substrate roughness due to the presence of the terminal thin disc [11]. Varenberg et al. found that detachment of the terminal disc happens from the inside out, with a peeling line moving from the center of the disc toward its outer edge [13]. In later work, Varenberg et al. reasoned that, as the terminal disc of mushroom-shaped micropillars detaches via a local thin-film peeling mechanism, multiple peeling fronts are present throughout the micropattern [14]. This splitting-up of the peeling front in multiple smaller fronts results in a drastic increase in peeling line length, and therefore in high pull-off and friction forces [14–15]. Heepe et al. investigated the significance of suction forces during detachment of mushroom-shaped micropillars [16], considering that the inside-towards-outside detachment mechanism gives rise to a low-pressure enclosed space in the center of the terminal disc during detachment. These authors empirically showed that suction forces are responsible for about 10% of the pull-off force mushroom micropatterns [16].

The presence of a terminal layer connecting neighboring micropillars at their tips has also shown to have a favorable effect on pull-off and friction forces on hard substrates. Glassmaker et al., for example, fabricated arrays of micropillars of 14 μm in diameter and 50 μm in height, where neighboring micropillars were connected at their tips with a continuous terminal layer of 4 μm in thickness [17]. These authors found that pull-off forces increased with increasing spacing between micropillars, and 9-times higher forces were generated compared to flat control samples at a spacing of 87 μm. The authors suggested that the increase in pull-off forces was caused by a crack-trapping mechanism during pulling off [17]. Bae et al. argued that the presence of a terminal layer leads to an increase of contact area with increasing preloads, resulting in higher pull-off forces under compression as compared to geometries without a terminal layer [18]. The friction of micropatterned adhesives with a terminal layer has been also investigated. He et al., for example, reported that, for a film-terminated ridge-channel structure, friction forces increased when channel width increased [19]. It was suggested that the terminal layer stretches during sliding, causing loss of elastic energy, thereby contributing to friction.

Besides geometry (i.e., shape), also the size of micropattern features has an effect on the *E*_eff_ of micropatterned adhesives. Varenberg et al. reasoned that finer micropillars have a lower contact stiffness, resulting in a lower contact reaction force, which might, in turn, result in higher pull-off forces, as long as the formed real contact area of the finer microstructure is not considerably lower than that of coarser microstructure [14]. Greiner et al. found that with increasing aspect ratio of micropattern features, their compliance increases, resulting in a better conformability to substrate roughness [20]. Hierarchical geometries, that is, architectures with features on different length scales, conform to substrate roughness on different length scales, increasing pull-off and friction forces [21].

Besides geometry and feature size, the *E*_eff_ of adhesive micropatterns also relates to the stiffness of the material the micropattern is made of [6]. When a soft material is used for the micropattern, the *E*_eff_ is low, leading to better defect control, stress distribution, and contact stiffness compared to micropatterns made of stiffer materials [22]. Also, the strength of the contacts formed between the adhesive and the substrate is affected by the material stiffness of the micropatterned adhesive, as this strength depends on the area of contact that is formed, which in turn is determined by the indentation depth of the adhesive into the substrate [23].

The performance of biomimetic micropatterned adhesives is usually tested on hard substrates, primarily glass and polystyrene. Much less is known about the performance of micropatterned adhesives when the substrate is deformable. Secure grip on soft, deformable substrates can be useful in a range of applications, including soft-tissue manipulation during surgical procedures and pick-and-place of soft biological objects such as grapes and poultry in food processing industries. The role of the geometry, feature size, and material stiffness of a micropattern on its pull-off and friction forces on a soft, deformable substrate can be expected to be different than on a hard substrate, as soft substrates deform under load and may conform to the geometry of the adhesive. For example, for a simplified representation of a discoidal adhesive element of a beetle, Heepe et al. showed that if the substrate is stiffer than the adhesive apparatus, a detachment mechanism similar to that observed for mushroom-shapes micropillars is present, with detachment starting from the center of the disc and moving toward its outer edge. However, if the substrate is softer than the adhesive apparatus, the latter potentially behaves like a flat punch, and detachment starts at the outer edge. Cheung et al. showed that during pulling off a micropattern from a soft substrate, the substrate deforms, and the detachment of neighboring pillars is no longer independent [24]. Accordingly, the pull-off force of mushroom-pillar micropatterns on a soft elastic substrate (Young’s modulus *E* = 200 kPa) has been found to be lower than on a rigid glass substrate [24].

On very soft substrates (Young’s modulus *E* ≈ 10 kPa), the indentation depth of microscale features is determined by a balance between the elastic properties of the substrate and the substrate–micropattern adhesion effects [25]. The length scale at which these adhesion effects are present is referred to as the elastocapillary length *l*, which is defined as *l* = γ/μ, where γ is the surface tension of the substrate and μ is the elastic shear modulus of the substrate [26]. If the length scale of the microscale features is in the order of the elastocapillary length, indentation is dominated by surface-tension effects, whereas for larger features, surface-tension effects are balanced by elasticity [25].

Summarizing, whereas for rigid substrates, adhesive micropatterns have been designed to gain a low *E*_eff_, it remains to be investigated whether this design approach should also be followed for adhesive micropatterns used on soft substrates. In order to gain insight into this question, we investigated the pull-off and friction forces of adhesive micropatterns on soft substrates as a function of the geometry and feature size of the micropattern, and the stiffness of both the substrate and the adhesive.

### Fabrication of micropatterned adhesives with various geometries, feature sizes, and stiffness degrees

Fabrication of micropatterned adhesives is most commonly done with molding techniques, in which a curable resin is shaped using a photolithographically fabricated three-dimensional hard template [3,24,27]. This fabrication method allows for the fabrication of a wide range of architectures and of features sizes at both nano- and microscale [28]. A limitation of this molding method is that demolding becomes challenging when the shaped material is soft. Another challenge of this method is that it requires complex instrumentation [28].

Akerboom et al. recently demonstrated a fast and cost-effective alternative method to fabricate micropatterns, in which a colloidal monolayer acts as a three-dimensional template to shape a curable resin [29–30], resulting in arrays of sub-microscale dimples [30]. This fabrication method allows for the demolding of resins even if, due to their softness, these adhere to the template, as demolding is done by chemically dissolving the colloidal template.

In this work, we used the abovementioned colloidal lithographic approach to fabricate adhesive micropatterns with various stiffness degrees. Moreover, we expanded the fabrication method in order to fabricate two dimple sizes: sub-microscale and microscale. Finally, considering the positive effect of a terminal layer on the adhesion of micropatterns, we expanded the fabrication process in order to also fabricate dimple arrays topped with a thin terminal layer.

The pull-off and friction forces of these micropatterns were tested on soft substrates made of poly(vinyl alcohol) (PVA) with two stiffness degrees and compared with the corresponding performance on glass as reference.

## Results

### Characterization of particles, micropatterns, and PVA substrates

The sub-microscale particles we used had an average diameter of 691 nm (SD = 14 nm), and a polydispersity index of 1.23. The average diameter of the microscale particles was 8.7 µm (SD = 1.4 µm), with a polydispersity index of 1.10.

**Figure 1:**
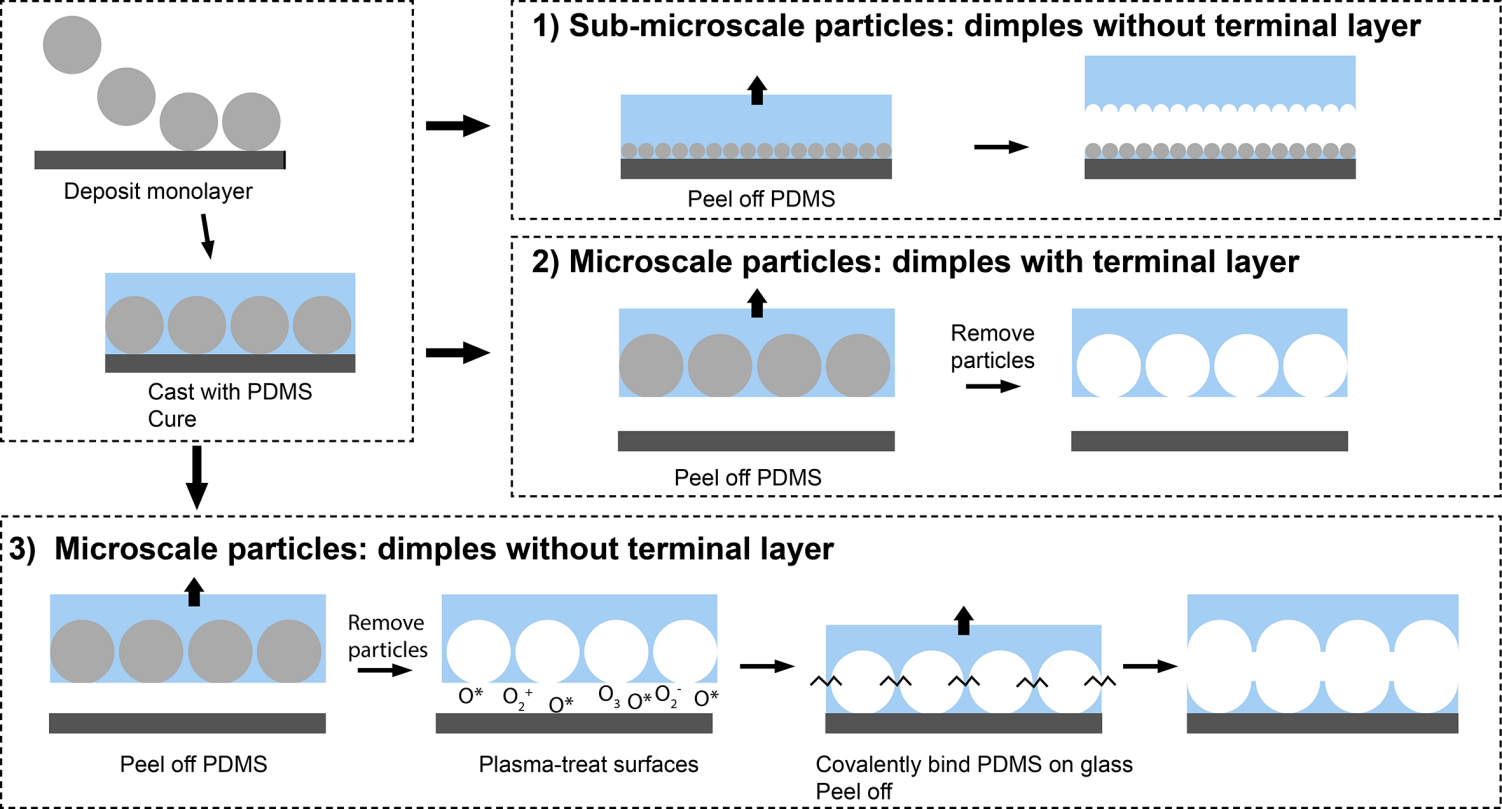
Pathway to fabricate dimple arrays with and without terminal layers. Starting from the left: deposition of a colloidal monolayer with a dip-coating cycle, followed by casting the monolayer with PDMS and subsequent curing. Depending on the particle size, the PDMS either comes off without the terminal layer (pathway 1), and the particles remain attached to the glass, or with the terminal layer (pathways 2 and 3), and the particles remain embedded in the PDMS. In the latter case, particles are subsequently removed by washing them in *N*-methyl-2-pyrrolidone. To obtain dimples without a terminal layer from microscale particles (pathway 3), dimples with a terminal layer from microscale particles are fabricated first, after which the terminal layer is removed by covalently binding it to glass, and subsequently peeling off.

**Figure 2:**
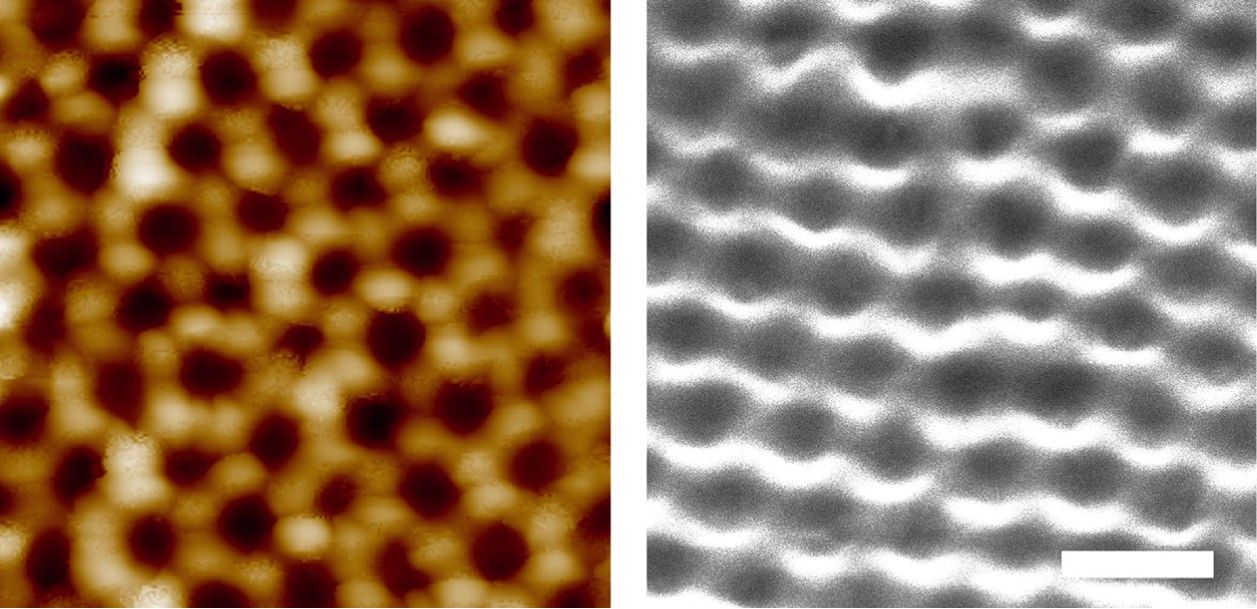
SEM and AFM images of micropatterns from sub-microscale particles. Left: Top view of the dimple micropattern after peeling off from untreated glass and removing the particles. A regular array of dimples is visible. Right: SEM picture of sub-microscale dimples. Charging of the edges of the micropattern impeded high-quality surface imaging. SEM data confirmed a homogeneous distribution of dimple packing and dimple size. The image was taken under an angle of 30°. The scale bar is 1 µm.

**Figure 3:**
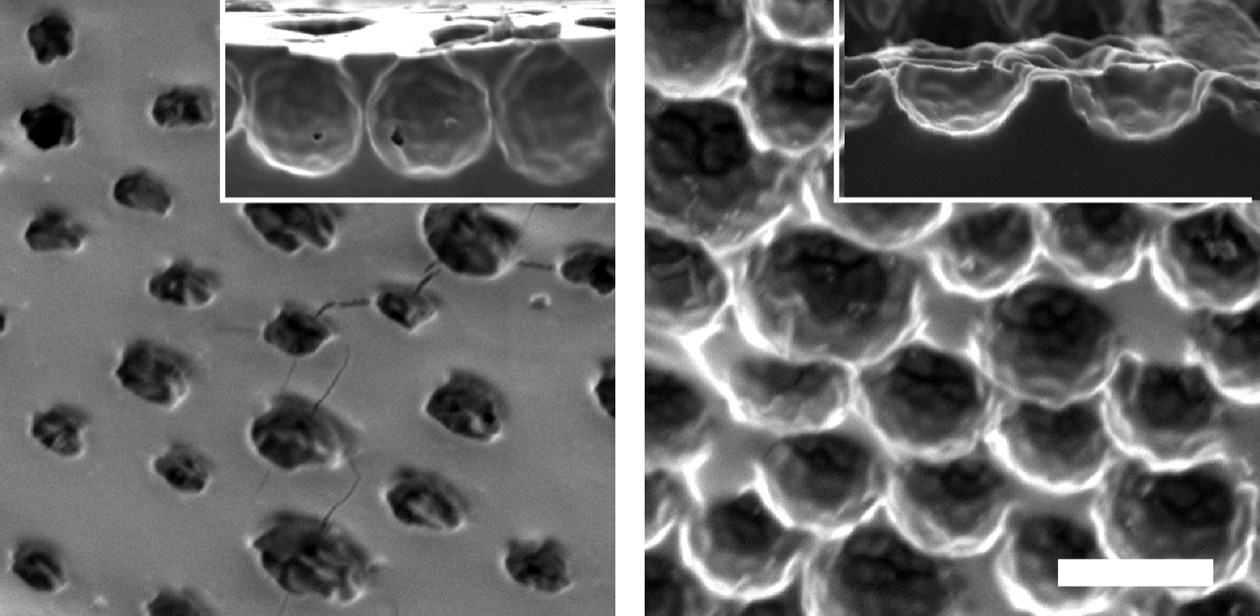
SEM images of micropatterns from microscale particles. Left: Micropattern with a terminal layer. The image was taken under an angle of 45°. Subsurface voids are visible through the holes; inset: Cross section of a micropattern with terminal layer showing spherical voids, separated by hourglass-shaped walls. Right: Array of dimples; inset: cross section of a dimple array showing a dimple depth of about 5 µm. The scale bar is 10 µm.

Micropatterned adhesives were fabricated from colloidal templates, as shown in Figure 1 and explained in the Experimental section. For the micropatterns of dimples from sub-microscale particles, the packing and size of the obtained dimples was homogeneous, as confirmed by AFM and SEM (Figure 2). AFM measurements further showed a dimple diameter of about 500 nm and a depth of about 200 nm (see section 2 of Supporting Information File 1, Figure S3). For micropatterns with dimples from microscale particles with and without a terminal layer, top view SEM images showed an average dimple diameter of 8.1 µm (SD = 1.17 µm, *n* = 100) (Figure 3, left). The depth of dimples from microscale particles could not be accurately determined from microscopic cross-section images, as it is unknown whether a dimple was sectioned through its center, where the diameter is largest. From the cross section shown at Figure 3 (left, inset), the dimple depth was equal to half of the dimple diameter. The cross section of samples with a terminal layer showed voids with a spherical shape, separated by hourglass-shaped walls (Figure 3, right, inset). These voids penetrate the terminal layer, resulting in an array of holes at the surface (Figure 3, right).

PDMS in 1:10 and 1:20 crosslinker/pre-polymer weight ratios was prepared, resulting in samples with Young’s moduli of 580 kPa (henceforth referred to as PDMS-580) and 280 kPa (PDMS-280), respectively [31].

The stiffness of the PVA substrates was adjusted by varying the number of freeze–thaw cycles. PVA subjected to two and three freeze–thaw cycles had storage moduli of 12 kPa (referred to as PVA-12) and 18 kPa (referred to as PVA-18), respectively, as measured using a rheometer (see section 1 of Supporting Information File 1). The dissipation factor tan δ was 0.05 and 0.07 for PVA-12 and PVA-18, respectively. The elastocapillary length of PVA (defined as *l* = γ_PVA_/μ_PVA_ [26], with surface tension γ_PVA_ ≈ 50 kPa [32] and elastic shear modulus μ_PVA_ ≈ 12 kPa for PVA-12) is in the order of 400 nm. Similarly, the elastocapillary length of PVA-18 is in the order of 300 nm.

### Pull-off forces of micropatterns on PVA and glass

**Figure 4:**
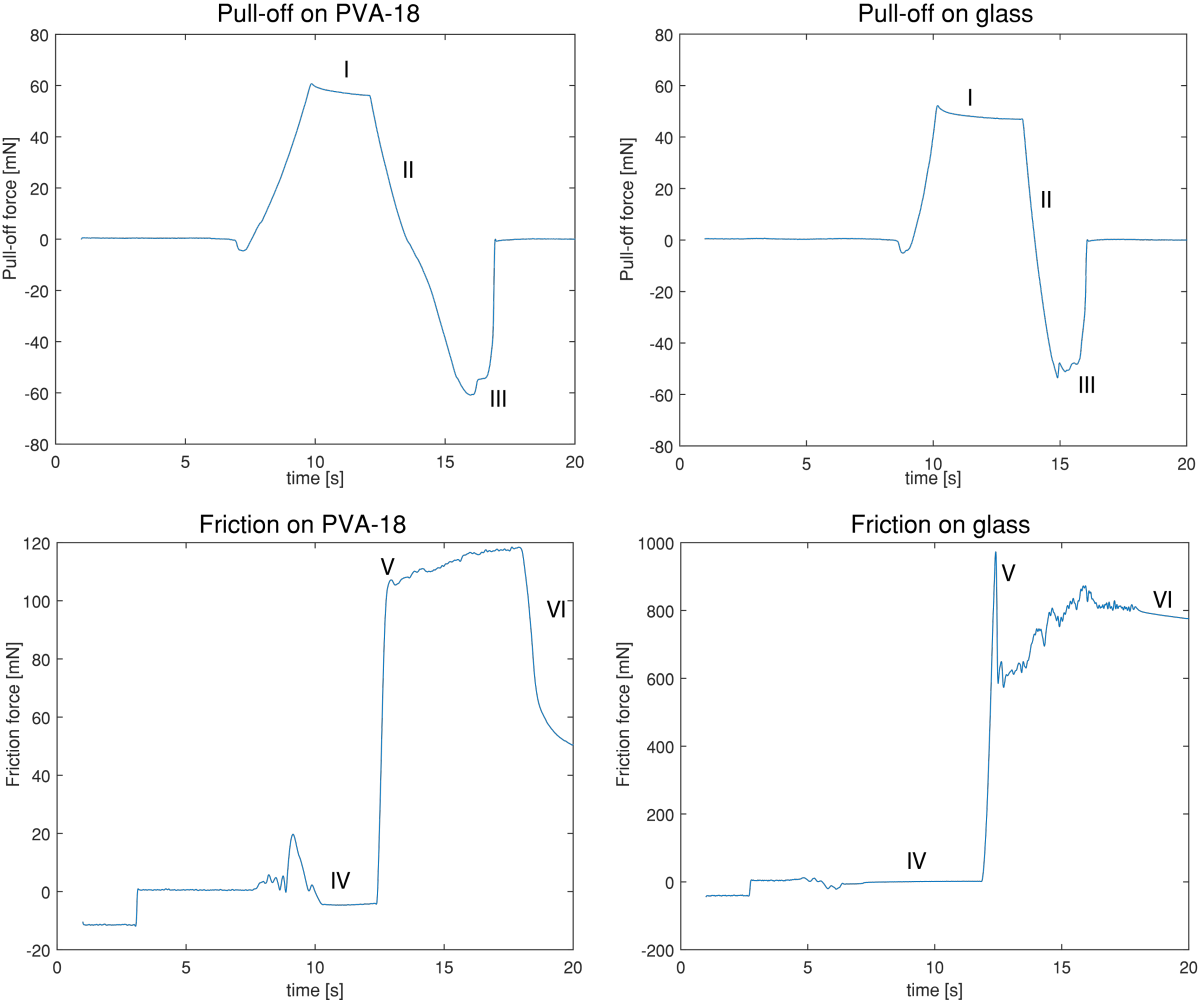
Representative force–time plots of pull-off force (top row) and friction (bottom row) measurements of microscale dimples without a terminal layer on PVA-18 (left column) and glass (right column). Pull-off force measurements: I) A normal preload of 55 mN is applied. II) The substrate is pulled off from the sample at 100 μm/s. III) The sample detaches from the substrate. The local minimum is reported as the pull-off force. Friction: IV) A normal (pre)load of 55 mN is applied. V) The substrate starts sliding at 500 μm/s. The first peak is reported as the static friction force. VI) After 6 s, sliding stops, and the forces in lateral direction decrease.

Figure 4 shows representative force–time plots of pull-off force measurements of microscale dimples without a terminal layer on PVA-18 and glass. It can be seen that detachment during pull-off (phase II) was slower on PVA than on glass.

**Figure 5:**
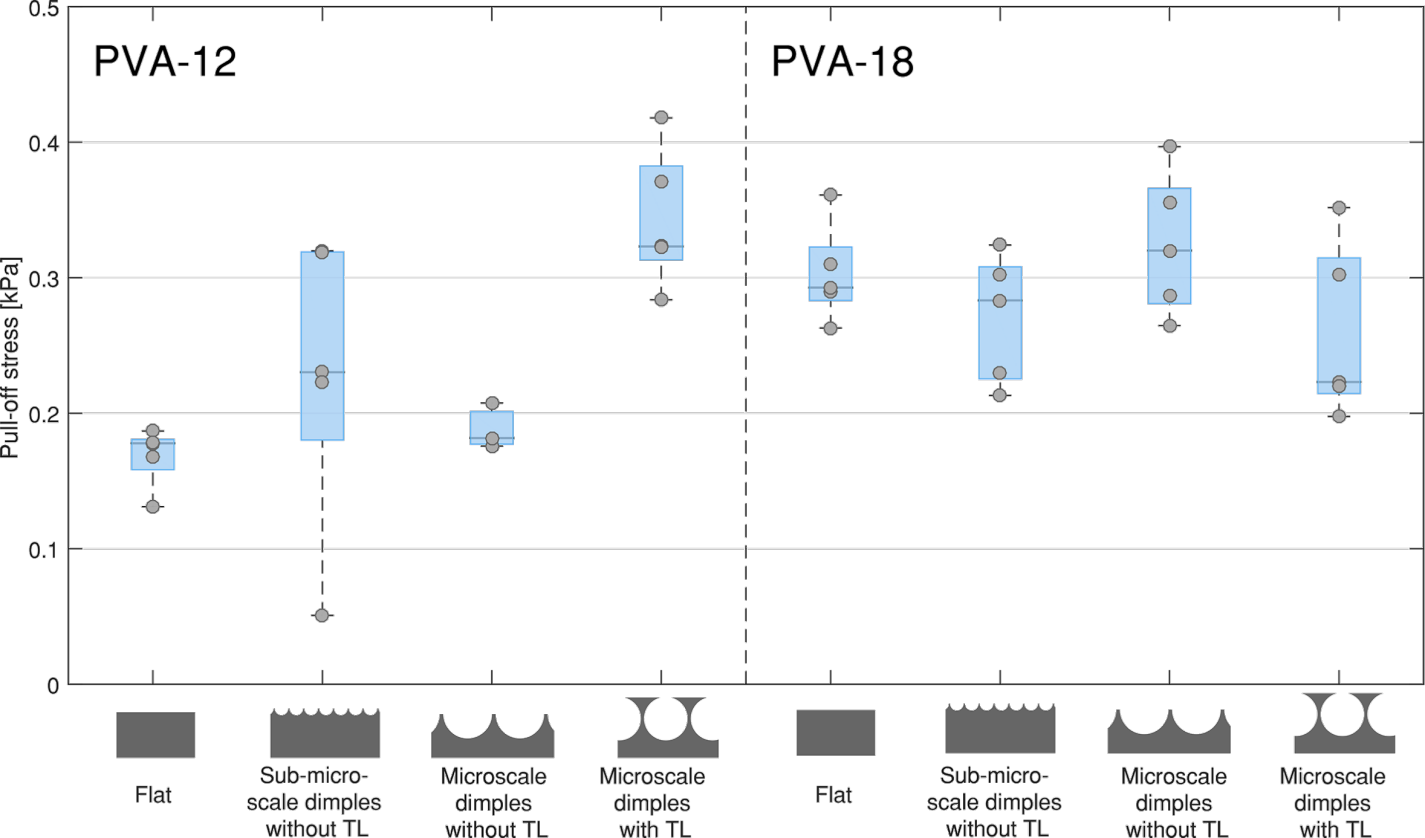
Pull-off stress (pull-off force divided by the sample area) for flat samples, sub-microscale dimples without terminal layer and microscale dimples with and without terminal layer, on PVA-12 (left) and PVA-18 (right). Only the results for PDMS-580 samples are shown. Each data point represents the average of five consecutive measurements of one sample, and each boxplot consists of five different samples of the same geometry.

Figure 5 shows the pull-off force on PVA-12 and PVA-18 normalized by the sample area (i.e., pull-off stress), for samples of PDMS-580 with various geometries and feature sizes. The results for the samples made of PDMS-280 are not shown here, as these exhibited similar trends to the PDMS-580 samples. Measurement data of PDMS-280 micropatterns, as well as descriptive statistics of the pull-off forces for all measured conditions are reported in section 5 of Supporting Information File 1.

A three-way ANOVA for sample geometry (flat, microscale dimples with terminal layer, and microscale dimples without terminal layer), sample stiffness (580 vs 280 kPa), and substrate stiffness (18 vs 12 kPa) showed significant main effects for the sample geometry (*F*(2,46) = 18.31, *p* < 0.001) and substrate stiffness (*F*(1,46) = 19.29, *p* < 0.001); the main effect of sample stiffness was not significant (*F*(1,46) = 2.32, *p* = 0.135). An interaction effect between sample geometry and substrate stiffness was also observed (*F*(2,46) = 29.61, *p* < 0.001). Post-hoc analysis showed that, on the softer PVA (PVA-12) and for both sample stiffness degrees, pull-off force of microscale dimples with a terminal layer was significantly higher than the pull-off force on flat samples as well as microscale dimples without a terminal layer (all *p* < 0.001 after Bonferroni correction). Flat samples and microscale dimples without a terminal layer did not exhibit significant difference in pull-off force (PDMS-580 samples: *p* = 1; PDMS-280 samples: *p* = 0.486). On the stiffer PVA (PVA-18), no significant effects of either sample geometry or sample stiffness were observed. Flat samples and microscale dimples without a terminal layer generated higher pull-off force on PVA-18 than on PVA-12 (PDMS-580 samples: both *p* < 0.001; PDMS-280 samples: both *p* = 0.003).

A three-way ANOVA for feature size (flat, sub-microscale dimples without terminal layer, and microscale dimples without terminal layer), sample stiffness (580 vs 280 kPa), and substrate stiffness (18 vs 12 kPa) showed a significant effect for substrate stiffness (*F*(1,47) = 32.63, *p* < 0.001); the main effects for feature size (*F*(2,47) = 2.78, *p* = 0.072) and sample stiffness (*F*(1,47) = 0.86, *p* = 0.358) were not significant. An interaction effect between feature size and substrate stiffness (*F*(2,47) = 10.2, *p* < 0.001) was also observed. Post-hoc analysis showed that pull-off force was significantly higher on PVA-18 than on PVA-12 for flat PDMS-580 samples and microscale PDMS-580 samples (*p* < 0.001). On PVA-18, microscale PDMS-280 samples exhibited significantly higher pull-off force than sub-microscale PDMS-280 samples (*p* < 0.001).

**Figure 6:**
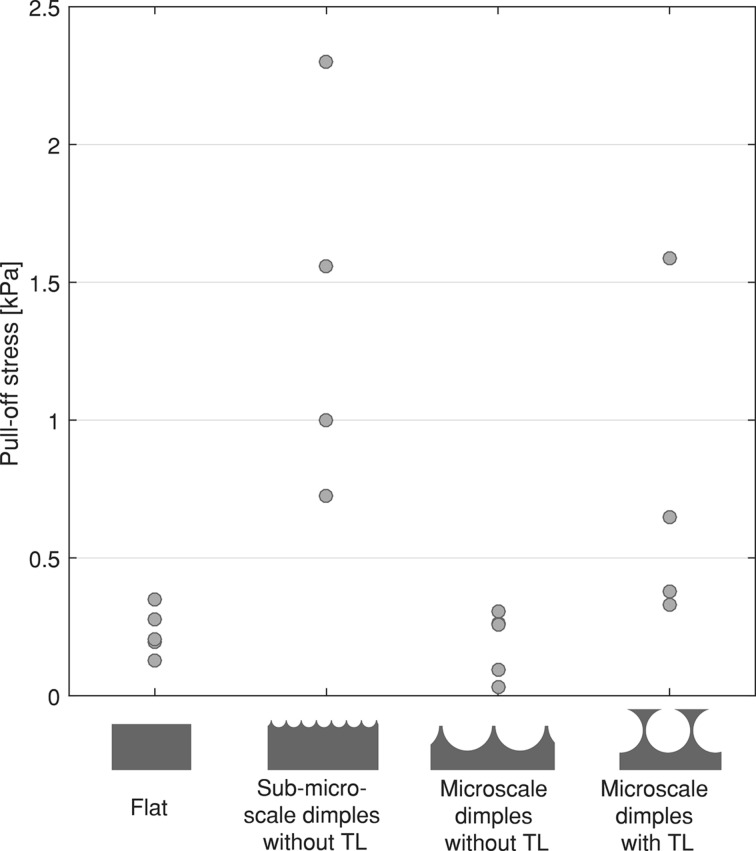
Pull-off stress for flat samples, sub-microscale samples without terminal layer and microscale samples with and without terminal layer on glass. Only the results for PDMS-580 samples are shown. Each data point represents the average of five consecutive measurements of one sample, and for each geometry, four or five samples have been tested. For sub-microscale samples without terminal layer and microscale samples with terminal layer (i.e., second and fourth geometry) one data point for each is missing because the measurement exceeded the maximum capacity of the sensor.

Figure 6 shows the pull-off stress on glass for samples of PDMS-580 with various geometries and feature sizes. The results for the samples made of PDMS-280 are not shown, as these exhibited similar trends to the PDMS-580 samples. It can be seen that sub-microscale samples and microscale samples with a terminal layer tend to generate higher pull-off forces than flat samples and microscale samples without a terminal layer. For these two conditions, one of the five measurements could not be completed because the sensor reached its maximum capacity. Because of the small sample size, we refrained from presenting boxplots with median and interquartile range, and present only raw data instead.

### Friction of micropatterns on PVA and glass

**Figure 7:**
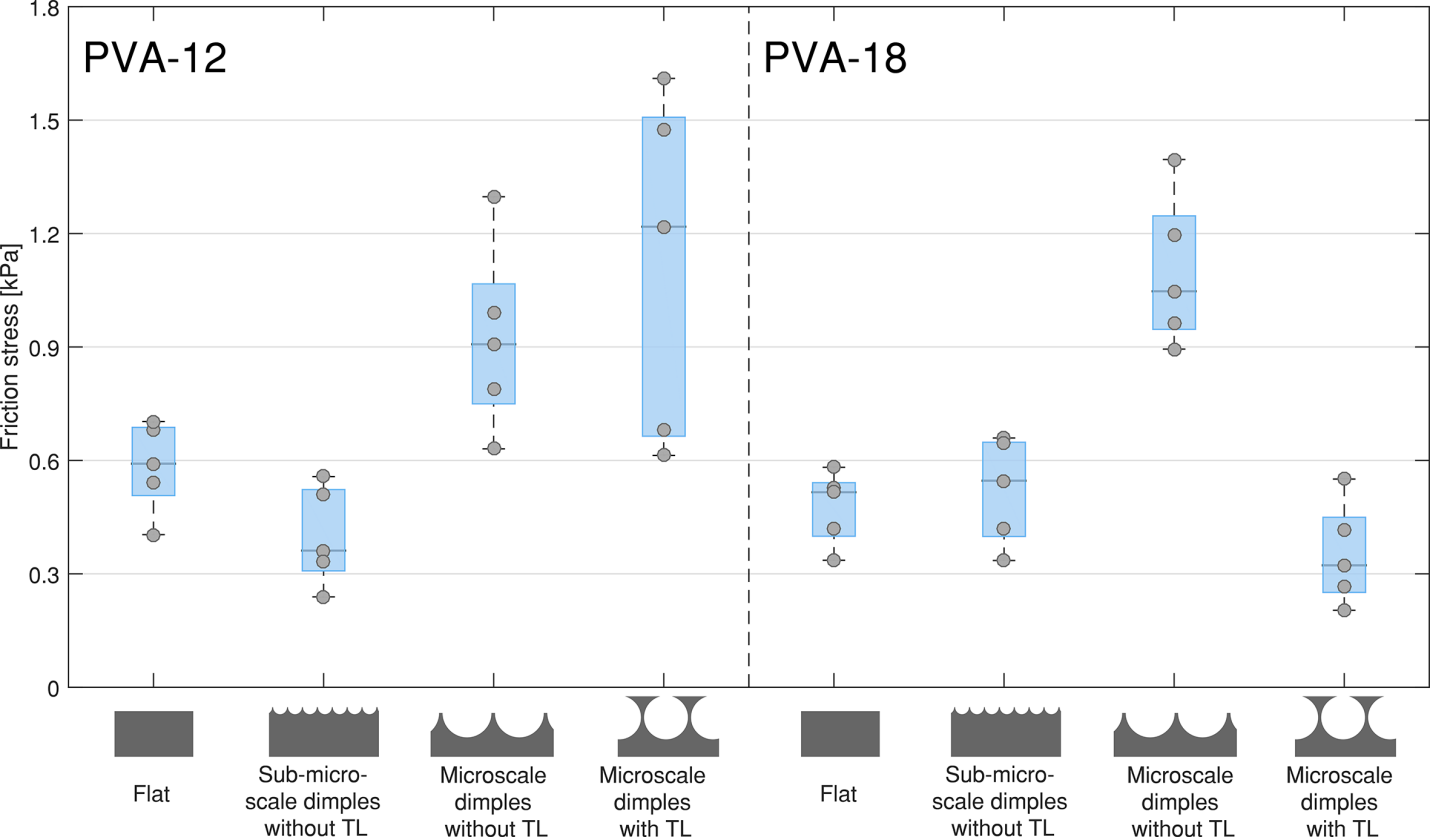
Friction stress (friction force divided by the sample area) for flat samples, sub-microscale samples without terminal layer, and microscale samples with and without terminal layer, on PVA-12 (left) and PVA-18 (right). Only the results for PDMS-580 samples are shown.

In Figure 4, time–force plots of friction measurements are depicted. Friction plots show that a static friction peak right before sliding (phase V) was observed only on glass but not on PVA. Figure 7 shows the friction forces on PVA-12 and PVA-18 normalized by the sample area (i.e., friction stress), for samples of PDMS-580 with various geometries and feature sizes. The results for the samples made of PDMS-280 are not shown, as these exhibited similar trends to the PDMS-580 samples. The results of friction measurements of all conditions are reported in section 5 of Supporting Information File 1.

A three-way ANOVA for sample geometry (flat, microscale with, and sub-microscale without terminal layer), sample stiffness (580 vs 280 kPa), and substrate stiffness (18 vs 12 kPa) showed significant main effects for the sample geometry (*F*(2,50) = 34.33, *p* < 0.001) and substrate stiffness (*F*(1,50) = 18.3, *p* < 0.001); the main effect of sample stiffness was not significant (*F*(1,50) = 0.09, *p* = 0.763). A small interaction effect between sample geometry and substrate stiffness was also observed (*F*(2,50) = 4.17, *p* = 0.021). Post-hoc analysis showed that on the harder PVA-18 substrate and for both PDMS-580 and PDMS-280, microscale samples without terminal layer generated higher friction than both flat samples (*p* < 0.001) and microscale samples with a terminal layer (PDMS-580: *p* < 0.001, PDMS-280: *p* = 0.031). The friction of the microscale samples with a terminal layer was not significantly different from the flat samples for either substrate and either sample stiffness.

A three-way ANOVA for feature size (flat, sub-microscale samples without terminal layer, and microscale without terminal layer), sample stiffness (580 vs 280 kPa), and substrate stiffness (18 vs 12 kPa) showed a significant effect for feature size (*F*(2,50) = 45.35, *p* < 0.001); the main effects for sample stiffness (*F*(1,50) = 2.43, *p* = 0.125) and substrate stiffness (*F*(1,50) = 3.00, *p* = 0.090) were not significant. A small interaction effect between feature size and sample stiffness was also observed (*F*(2,50) = 7.39, *p* = 0.002). Post-hoc analysis showed that friction was significantly higher for microscale samples than for flat samples for both sample stiffness degrees and both substrate stiffness degrees, with the effect being stronger for the softer substrate (PVA-12: PDMS-580, *p* = 0.001, PDMS-280, *p* = 0.003; PVA-18: both *p* < 0.001). Microscale samples also generated higher friction than sub-microscale samples for PDMS-580 (PVA-12: *p* < 0.001, PVA-18: *p* = 0.002), whereas for PDMS-280 both sub-microscale and microscale samples generated equally high friction.

**Figure 8:**
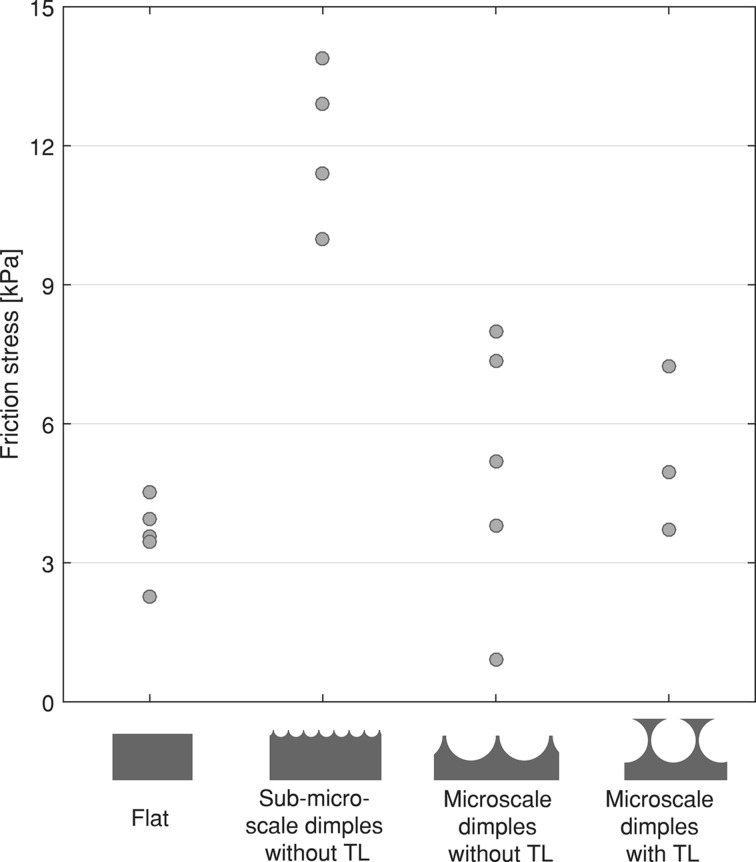
Friction stress for flat samples, sub-microscale samples without terminal layer, and microscale samples with and without terminal layer, on glass. Only the results for PDMS-580 samples are shown. One data point for each geometry is missing because these measurements exceeded the maximum capacity of the sensor.

Figure 8 shows the friction stress on glass for samples of PDMS-580 with various geometries and feature sizes. Sub-microscale samples without terminal layer seem to generate higher friction than the remainder of the samples, but we refrain from drawing any conclusions, as for 6 out of the 35 measurements the sensor reached its maximum capacity (for PDMS-580: one measurement for each of the four samples; for PDMS-280: two measurements for micrometer samples with terminal layer).

## Discussion

In this work, we expanded a recently introduced colloidal lithographic approach and showed that it is possible to fabricate micropatterns with microscale dimples that are about one order of magnitude larger than the (sub-)micrometer-sized dimples reported in [28,30,33–34], with stiffness values down to 280 kPa, which is lower than the typical stiffness in the megapascal-range achieved by soft molding [35]. This fabrication method showed to be highly repeatable, and provided consistent results in terms of geometrical properties. With this fabrication method, we also demonstrated how to fabricate dimple arrays with and without a terminal layer. The pull-off and friction forces of the fabricated micropatterns were measured on soft substrates as a function of feature size, stiffness degree of the micropattern and of the substrate, and the presence or absence of a terminal layer.

### Pull-off forces

#### Effect of geometry and stiffness on pull-off forces on soft substrates

Pull-off measurements on soft substrates show that micropatterns of sub-microscale and microscale dimples without a terminal layer do not generate significantly higher pull-off forces than flat samples. We assume that, for both dimple sizes, the soft substrate fully conforms to the dimples, and the formation of independent contacts does not happen. Sub-microscale dimples have a depth of around 250 nm. As the elastocapillary length of PVA substrates is in the order of 400 nm, the PVA substrates fully conform to the micropattern based on surface tension effects, without elastic penalty. Microscale dimples have a dimple depth of around 5 μm, which is well above the elastocapillary length of PVA, and conformation to the micropattern is expected to be elastically dominated. As a result of the conformation properties of the substrate, a single larger contact area is formed, and advantageous effects of defect control and crack trapping mechanisms, as reported for rigid substrates [30], are not present.

A microscale dimple geometry with a terminal layer generated higher pull-off forces compared to other tested geometries and flat control samples on the softer PVA substrate (PVA-12). A possible underlying mechanism explaining the positive effect of the terminal layer on pull-off force is that the soft PVA substrate interlocks with the holes of the terminal layer. Deformation of the PVA substrate, resulting in protrusions perforating the terminal layer, is elastically dominated, as the terminal layer thickness is well above the elastocapillary length of PVA of 400 nm. Formation of protrusions is a trade-off between, on the one hand, elastic stresses and, on the other hand, the compressive load on the bulk. On the stiffer PVA-18 substrate, this positive effect of a terminal layer on pull-off forces was not observed. PVA-18 has a higher elasticity, likely resulting in a higher elastic penalty for protrusion formation than in the case of the PVA-12 substrate. Therefore, during pulling off, formed protrusions jump back, and interlocking is lost faster on the PVA-18 substrate compared to the softer PVA-12 substrate. We expect that crack trapping mechanisms, as reported for terminal-layer geometries on hard substrates, are not involved on the tested PDMS-PVA configurations. As Heepe et al. already reasoned for a (simplified) representation of a discoidal adhesive element [6], the advantageous effect of a thin film micropattern on pull-off force is lost when the substrate is soft compared to the adhesive.

Suction forces might also play a role in generating grip with arrays of dimples, both with and without a terminal layer. Air in dimples or, in the presence of a terminal layer, in the sub-surface cavities, will be squeezed out during loading, resulting in suction during detachment. We do not expect that suction is a dominant mechanism in the tested micropatterned adhesives, as there was no significant difference in pull-off forces between sub-microscale and microscale dimples without a terminal layer on soft substrates, despite the fact that sub-microscale dimples have a much lower suction cup volume compared to microscale dimples. Spolenak et al. found that at contact radii smaller than 10 μm, as is the case for our geometries, suction cups rapidly lose their effectiveness [36].

Force–time plots of pull-off force on soft substrates (Figure 4) show that during pulling off (phase II in Figure 4), the drop in force took a few seconds longer compared to pulling off from glass substrates, indicating that contact was lost less abruptly on soft substrates. This gradual contact loss is probably caused by deformation of the soft substrate during pull off, as observed by Cheung et al. [24]. We did not test whether this deformation has a dissipative or an elastic nature, a question that could be investigated in future works by varying the pull-off speed. Force–time plots on soft substrates also show that the peak force at phase III was wider compared to measurements on glass, indicating that detachment from PVA was slower than from glass.

On soft substrates, we did not find a consistent effect of the theoretical contact area of the measured geometries on pull-off force. For example, while microscale dimples without a terminal layer have a higher contact area compared to sub-microscale dimples without a terminal layer, the former did not generate higher pull-off forces compared to the latter on soft substrates. This observation might indicate that the contact formed between micropattern and substrate is not a strong contact. A low strength of the formed contact might be explained by PVA having a low surface energy (ca. 50 mN/m [32]), and because of the presence of water at the PVA–micropattern interface, which might be squeezed out of the PVA gel during loading.

Whereas geometry did not show consistent effects on pull-off force, the substrate stiffness did exhibit a systematic effect on pull-off forces for geometries without a terminal layer and for flat control samples, generating higher pull-off forces on the stiffer PVA-18 substrate compared to the softer PVA-12. This result is logical, because, given that the PVA substrates are much softer than the used microstructures (*G*′_PVA_ ≈ 10^1^ kPa; *E*_PDMS_ ≈ 10^2^ kPa), the substrate is expected to be the main component to deform when stress is applied.

Geometry effects, if present, are unlikely to significantly contribute to the generated pull-off forces and friction forces, because the soft substrates likely fully conform to the micropattern. The PVA substrates have some dissipative properties (dissipation factors of PVA-12: tan δ = 0.05; PVA-18: tan δ = 0.07), which might contribute to the resultant pull-off force as well. Given the low value of these dissipation factors, we doubt whether damping plays a significant role in generated pull-off forces.

Our measurement data suggest that, when the substrate is softer than the adhesive, the substrate conforms to the features of the adhesive when load is applied, enabling intimate contact [37–38]. The intimate contact has a positive effect on pull-off and friction forces, as long as the elastic penalty of the substrate deformation does not dominate over surface energy effects. Because the formed intimate contact between a micropatterned adhesive and a conformed soft substrate is a singular contact, geometry-induced defect control and stress distribution are not expected to be present on a soft substrate when the adhesive micropattern is stiff compared to the substrate.

#### Effect of geometry on pull-off forces on hard substrates

Measurements on glass showed that sub-microscale samples tend to generate higher pull-off forces than flat samples and microscale samples without a terminal layer and flat samples. Crack trapping, as proposed for similar microscale dimple arrays by Akerboom et al. [30], is likely more dominant in the smaller (sub-microscale) features than in the microscale micropatterns. Furthermore, sub-microscale dimples might form complete contact with the substrate [30], generating a higher contact area compared to other geometries. Because of the high surface energy of glass (about 1000 mJ/m^2^ [39]), the formed contact points between the micropattern and the substrate are stronger than the contact points between micropattern and PVA substrates, which may partially explain the higher pull-off forces on glass compared to soft substrates.

Microscale dimples without a terminal layer did not generate higher pull-off forces compared to flat control samples. We expect that, under the applied load, the elastic penalty for making full contact dominates over the gained pull-off force as a result of formed contact for this geometry.

Similar to the results on the soft substrates, microscale dimples with a terminal layer tended to generate higher adhesive forces on glass compared to the same dimples without a terminal layer and flat samples. In line with Glassmaker et al. [17], we assume that a crack-trapping mechanism plays a role in our terminal-layer geometries. Additionally, crack trapping may be promoted by the presence of microscale voids in the terminal layer, similar to the observations by Hwang et al., who found enhanced pull-off forces by using cuts in the applied materials, thereby introducing compliant regions in stiff adhesive films [40]. The presence of a terminal layer further enhances pull-off forces because of the deformability of the former, resulting in a higher effective contact area than micropatterns without a terminal layer [17]. This deformation effect of the terminal layer on pull-off force is supported by the findings by Shahsavan et al., who reported that with thin film-terminated micropillars higher compliance and pull-off forces can be realized when the terminal layer has viscoelastic material properties [41]. For microstructures of dimples with a terminal layer, deformation of the terminal layer is likely to happen, given that the elastic modulus of PDMS is in the kilopascal-range, and thus elastic, and the thickness of the terminal layer is limited (i.e., conformation to substrate roughness requires only a small volume of material to elastically deform, resulting in a minor elastic penalty for conformation). The result that higher pull-off forces are generated with the softer PDMS-280 microstructures compared to PDMS-580 microstructures supports a deformation effect of the terminal layer. Besides elastic stretching of the terminal layer, the effective modulus of the dimples with terminal layer is likely lower compared to other geometries, because of the presence of sub-surface voids.

A suction mechanism, if present, is expected to play a more dominant role on the rigid and impermeable substrate of glass than on PVA substrates [33]. However, we do not expect that suction forces are the main mechanism generating pull-off forces in the tested geometries, as sub-microscale dimples, despite having much smaller suction cups compared to microscale dimples, outperformed microscale dimples on glass.

### Friction forces

#### Effect of geometry and stiffness on friction forces on soft substrates

On soft substrates, force–time plots of friction force (Figure 4) show that the static friction force (phase V in Figure 4) is comparable to the dynamic friction. A minor increase in friction force during sliding was typically observed, presumably caused by the PVA substrate “piling up” at the front line during sliding of the micropattern. On the stiffer PVA (PVA-18) substrate, large dimples without a terminal layer outperformed all other geometries. A similar, albeit less pronounced, effect was also observed on the softer PVA-12 substrate. We assume that with large dimples indent deeply into the PVA substrates, generating mechanical interlocking and a relatively high contact area. The microstructure starts moving when this interlocking is lost due to deformation of the substrate. A low indentation depth, as it is expected for flat samples, sub-microscale dimples and dimples with a terminal layer, requires a smaller volume of substrate to elastically deform to start sliding, resulting in lower friction forces. On the softer substrate of PVA-12, the elastic penalty for deforming is lower compared to PVA-18, which can explain why the superior performance of microscale dimples without a terminal layer on PVA-18 was less pronounced on the softer PVA-12.

Dimples with a terminal layer generated higher friction on the softer substrate of PVA-12 compared to the stiffer PVA-18, in line with the findings for pull-off force measurements. It is possible that the same protrusion formation as described for pull-off force measurements also holds for friction measurements, with the substrate protruding into the sub-surface voids of the microstructure. Similar to pull-off experiments, suction forces cannot be ruled out either.

#### Effect of geometry on friction forces on glass

On the glass substrate, force–time plots of friction force (Figure 6) show that static friction (peak at phase V in Figure 6) is dominant over dynamic friction. Some sort of zigzag was typically visible in the dynamic friction regime, indicating stick-slip-like behavior during sliding for both flat and micropatterned samples.

Our results suggest that sub-microscale dimples led to higher friction forces compared to flat samples and to large dimples with or without terminal layer. We expect that under the applied preload, sub-microscale dimples flatten, and a contact area similar to flat samples is formed. Due to stored elastic energy in the micropattern, the formed contact might be better preserved during sliding compared to a flat geometry, resulting in higher friction forces.

For a microscale dimple geometry without a terminal layer, friction forces are similar to or even lower than the friction forces of flat control samples on glass. Similar to the pull-off force measurements, we assume that the applied load during sliding is not sufficient to bring the bottom of the dimples into contact with glass, leading to a small contact area and thus low friction forces.

Microscale dimples with a terminal layer generate higher friction forces compared to flat control samples. This might be related to the compliance of the terminal layer, due to which the contact during sliding is more efficiently conserved compared to flat samples. Elastic storage by means of stretching of the terminal layer, as suggested by He et al. [19], might also occur, leading to an increase in friction. Besides, as already noted earlier, because of the presence of spherical voids below the surface, the effective modulus of the terminal-layer micropatterns is likely lower compared to other geometries and flat control samples.

### Limitations and recommendations for future work

In our experimental setup, we performed pull-off and friction measurements in a plate-to-plate configuration. We took extensive measures to assure proper alignment of the sample on the substrate, including visual inspection of the sample–substrate interface prior and during measurements using a magnifying camera, and real-time inspection of the recorded time–force curves. Moreover, the platform on which the substrate was placed was positioned between three sets of springs (flexures), which gave the platform some self-aligning properties. Despite these measures, we suspect that the high variation of the measurement data on glass was caused by misalignment.

To counterbalance such issues of misalignment, our experimental design and statistical analysis were conservative: each data point was the average of five consecutive repeats and the measurements of independent samples were done in a randomized order. We also opted for a low α value of 0.001. It should be further noted that the increase in random variance because of misalignment and other side effects was not too large to dilute the strongly significant non-random effects we observed. On soft substrates, the variation of the measurement data was lower, which is logical, because the flexibility of the soft substrate ensures that the sample establishes good contact with the substrate. For follow-up experiments, the use of a (hemi-)spherical probe instead of a plate-to-plate configuration can be considered, to avoid misalignment issues.

Due to the limited force range of our measuring setup, some samples could not be measured on glass. Considering the limited amount of data, we refrained from drawing conclusions on the effect of microscale samples with and without a terminal layer on friction.

The fabricated sub-microscale dimples had a lower depth than the particle radius. Considering that the time between casting the monolayer with PDMS, degassing and subsequent curing at 68 °C was in the order of 15 min, the uncured PDMS does not fully flow through the colloidal monolayer on this timescale, resulting in a limited dimple depth. A strategy to increase the PDMS penetration into the monolayer would be to cure the PDMS at room temperature for 48 h, in which case PDMS remains in a liquid state for much longer. Indeed, we did observe larger dimples and thinner walls between dimples when curing the sample at room temperature in a post-hoc synthesis, as can be seen in section 3 of Supporting Information File 1.

Given the high pull-off and friction forces of microscale dimples with a terminal layer on both hard and soft substrates, it would be interesting to test the performance of sub-microscale dimples with a terminal layer. However, we were not able to fabricate sub-microscale dimples with a terminal layer, presumably because the walls between dimples are so thin that they break during peeling off from the template, or because the uncured PDMS did not fully penetrate the monolayer. The latter problem could be solved by creating colloidal monolayers with a larger spacing, for example by optimizing the surface chemistry of particles.

The mechanism of generating grip on the tested substrates is likely indentation-based, creating mechanical interlocking, and therefore strongly depends on the stiffness of both substrate and adhesive. Consequently, it is not surprising that our results pointed towards higher friction on soft substrates when employing large dimples compared to small dimples. This result suggests that with even larger dimples the friction performance of micropatterns on soft substrates can be improved, even under low (pre)loads, a hypothesis that deems further investigation.

In our work, the stiffness of the substrate was much lower than the stiffness of the sample. Future work could be directed towards testing configurations in which the stiffness of adhesive and substrate are of the same order. Our hypothesis is that in this case, contact loss due to substrate deformations is prevented, and effects of geometry, such as increased contact area with a dimples-with-terminal-layer geometry, become visible. Although the use of a much softer micropattern might give rise to geometry effects, it remains to be investigated whether the loss in contact strength accumulatively leads to an increase in pull-off force.

While we found a significant effect of the geometry on pull-off and friction forces on soft substrates, it was difficult to clarify the underlying mechanisms that cause these effects, both qualitatively and quantitatively. The hypothesized interlocking effects could be investigated in future studies by quickly freezing microstructure–substrate complexes when under load and studying their cross section with optical microscopy. The importance of deformation mechanisms of the substrate in the pull-off and sliding of our adhesives could be further investigated by varying the pull-off or sliding speed, since the strain rates of both substrate and adhesives are time dependent.

## Conclusion

We used a facile, out-of-the-cleanroom method to fabricate microstructures with sub-microscale features, and expanded it for microscale features. We fabricated geometries of moderate architectural complexity (extruded patterns with curved surfaces) and of high architectural complexity (overhanging features), at different length scales and different degrees of stiffness.

We found that higher pull-off and friction forces on soft substrates were generated with larger feature sizes. On soft substrates, the positive effects of sub-microscale features on pull-off and friction forces, such as defect control and crack trapping, are not present, because the substrate conforms to the micropattern. Instead, interlocking is likely the dominant mechanism of pull-off and friction forces on soft substrates.

The effect of the microstructure stiffness was not pronounced, which is not surprising, considering that the microstructures were one order of magnitude stiffer than the soft substrate, meaning that the latter was the main component to deform. We expect that the effect of the microstructure stiffness becomes larger when it is in the same order as the substrate stiffness, in which case both the microstructure and the substrate compete to deform.

In conclusion, we found that, on soft substrates, microscale dimples generate higher pull-off and friction forces than sub-microscale dimples. Generation of grip on soft substrate seems to be dominated by different underlying mechanisms than those holding for hard substrates.

## Experimental

### Materials

Sylgard-184 pre-polymer (base) and crosslinker (curing agent) were purchased from Dow Corning, poly(vinyl alcohol) (PVA, Selvol PVOH 165; hydrolysis rate: 99.65% ± 0.35%; degree of polymerization: about 2000, as reported by the manufacturer) was purchased from Sekisui Chemical Group. *N*-methyl-2-pyrrolidone (NMP) was purchased from Sigma-Aldrich. DVB/Sulfate latex particles with a reported diameter of 10 µm were purchased from ThermoFisher Scientific as a 4 % (w/v) dispersion in water and were dispersed in ethanol to get an 8% w/v dispersion before use.

### Synthesis and characterization of particles

**Sub-microscale particles:** Carboxylated polystyrene (PS) particles with a sub-microscale diameter were synthesized in a single-step surfactant-free emulsion polymerization, according to Appel et al. [42]. The particles were washed by centrifugation three times in ethanol and three times in water. The particles were dispersed in ethanol to obtain a 20% (w/v) dispersion before use. Particle size and polydispersity index were determined with a Malvern Nano ZS 3600 Zetasizer. The laser had a wavelength of 633 nm and a scattering angle of 173°.

**Microscale particles:** The purchased microscale particles were characterized by assessing microscopic images of dispersion droplets of particles in water. Diameters of 100 particles were determined using ImageJ [43], and the average diameter and polydispersity index were determined using equations 1–3 from Nematollahzadeh et al.[44].

### Fabrication of micropatterns

**Deposition of colloidal monolayers on glass using dip coating:** Colloidal monolayers from sub-microscale and microscale particles were obtained by deposition of particles on an untreated microscopic slide of glass (75 × 26 mm^2^) (Corning^®^) using a dip coating process [45]. Specifically, a Langmuir–Blodgett trough (KSV Nima KN2002, medium-sized) was filled with demineralized water, and the microscopic glass slide was partially immersed for 20 mm in the bath in vertical direction. A plasma-treated glass cover slip was placed in the filled trough against one of the barriers in a diagonal orientation. The particle dispersion was added dropwise via the glass cover slip. Particles were added until a nearly packed monolayer was observed. Surface pressure was measured using a Wilhelmy plate.

After complete evaporation of the ethanol was achieved, as confirmed by stabilization of the surface pressure, the monolayer was compressed by moving the barriers toward each other until a sharp increase in surface pressure was observed, indicating close packing of the colloidal monolayer. A single dip-coating cycle was done by pulling out the glass slide vertically at a speed of 0.5 mm/s while keeping the surface pressure constant.

**Dimples without a terminal layer from sub-microscale particles:** Samples with dimples from sub-microscale particles were fabricated according to pathway 1 shown in Figure 1. Uncured pre-polymer/crosslinker mixture (henceforth referred to as uncured PDMS) was degassed in a desiccator, and cast on a 14 × 14 mm^2^ area of the monolayer, obtaining a thickness of 4 mm (see also section 4 of Supporting Information File 1). The monolayer with cast PDMS was placed in an oven for 2 h at 68.3 °C to cure the PDMS. The cured PDMS was peeled off from the glass slide, leaving the monolayer attached to the glass together with a terminal PDMS layer. Following Akerboom et al. [30], residual particles were removed from the sample by cleaning it with Scotch Magic Tape, and by immersing it in NMP for 1 h under stirring. Subsequently, while still immersed in NMP, the sample was placed in an ultrasonic bath for 1 min.

**Dimples with a terminal layer from microscale particles:** Samples with dimples from microscale particles and with a terminal layer were fabricated by casting uncured PDMS on a 14 × 14 mm^2^ area of the monolayer, with a thickness of 4 mm, and by subsequently curing it in an oven for 2 h at 68 °C (see also section 4 of Supporting Information File 1). Opposite to the case of sub-microscale particles described in the previous paragraph, in which peeling off the cured PDMS left both the monolayer and a terminal PDMS layer attached to the glass, upon peeling off the cured PDMS from the glass slide with microscale particles, the monolayer remained embedded in the PDMS and the terminal PDMS layer came off from the glass surface (see pathway 2 in Figure 1). The sample was washed to dissolve the monolayer by immersing it in NMP for 1 h under stirring. Subsequently, while still immersed in NMP, the sample was placed in an ultrasonic bath for 1 min.

**Dimples without a terminal layer from microscale particles:** Dimple arrays without a terminal layer from microscale particles were fabricated according to pathway 3 in Figure 1. Dimple arrays with a terminal layer were first fabricated as described in the previous paragraph. Then, the samples were covalently attached to a glass slide by plasma-treating both the glass and the sample surfaces, and bringing the treated surfaces together. After applying some load, the sample-on-glass was placed in an oven for 20 min at 68 °C to form covalent bonds between the two. After binding, the sample was peeled off from the glass slide. Upon peeling off, the terminal layer remained attached to the glass slide. The peeled-off sample separated from the terminal layer, resulting in a micropattern with dimples.

All three types of samples (1: dimples without a terminal layer from sub-microscale particles; 2: dimples with a terminal layer from microscale particles; and 3: dimples without a terminal layer from microscale particles) were prepared using two crosslinker/pre-polymer weight ratios, namely 1:10 and 1:20.

Flat samples were also fabricated as controls. To do so, we degassed uncured PDMS of 1:10 and 1:20 crosslinker/pre-polymer weight ratios in a desiccator. Uncured PDMS was cast on a 14 × 14 mm^2^ area of an untreated microscopic glass to obtain a layer of 4 mm thickness, and subsequently cured in an oven for 2 h at 68 °C.

### Characterization of micropatterns

Monolayers and samples from sub-microscale particles were characterized with atomic force microscopy (AFM), optical microscopy, and scanning electron microscopy (SEM). Monolayers and samples from microscale particles were characterized with optical microscopy and SEM. The elastic modulus of the fabricated micropatterns was measured with a TA Instruments AG-2R rheometer. A parallel-plate geometry with a diameter of 25 mm was used. Storage and loss moduli were determined at a strain of 0.05%, for a frequency range from 1·10^−1^ to 1·10^2^ rad/s, as can be seen in Supporting Information File 1 (Figure S1 and Figure S2). We use the storage moduli *G’* as measured at an angular velocity of 0.1 rad/s, since the pull-off and friction measurements are done at similar velocities.

### Fabrication and characterization of poly(vinyl alcohol) substrates

Poly(vinyl alcohol) (PVA) substrates were fabricated by filling 3D-printed molds with 10% (w/v) PVA hydrogel. In a PVA gel, crosslinks between chains are formed by hydrogen bonding between hydroxyl side groups. We used hydrolyzed PVA, because by additional hydrolysis, acetate side groups in the polymer are turned into hydroxyl groups, and crosslink formation is promoted. Subjecting PVA to freeze–thaw cycles further stiffens the hydrogel by growing local crystalline regions that act as network junctions [46]. We prepared substrates of two stiffness degrees, by subjecting PVA to two or three freeze–thaw cycles, respectively.

### Measuring pull-off and friction forces

**Figure 9:**
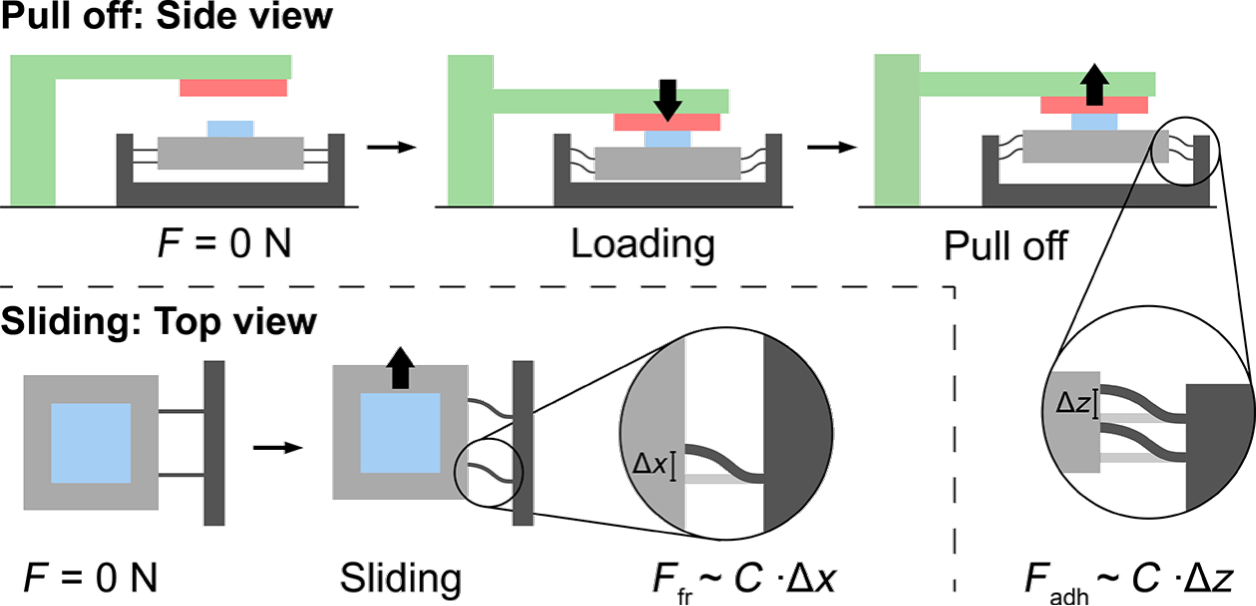
Schematic representation of the customized measuring setup in the configuration of a pull-off force measurement (top line) and a friction measurement (bottom line). Pull-off: The micropatterned adhesive (blue) is mounted on a holder (grey) suspended via three sets of parallelogram-flexures. The substrate (red) is brought in contact with the sample using a translation stage (green). When the substrate is pulled off, forces are exerted on the sample holder, which gets displaced vertically. The pull-off force is calculated from the holder displacement Δ*z* via the flexure stiffness *C*. Friction: The substrate (transparent, red) is brought in contact with the micropattern, and the substrate is displaced laterally. Before the micropattern starts sliding, the force platform is displaced in lateral direction. The holder displacement Δ*x* at the moment the micropattern starts sliding is recorded, and the friction force is calculated from the holder displacement Δ*x* via the flexure stiffness *C*.

Pull-off and friction forces of the samples were measured with a custom-built force transducer (see Figure 9 for a schematic representation of the setup). The force transducer consisted of a sample holder suspended via three sets of serially arranged parallelogram-flexures which allowed for the translation of the sample in three orthogonal directions. The displacement of the sample holder in the three directions was measured with confocal chromatic aberration sensors (CL1 MG210; Stil S.A.S) controlled with Prima controllers (Stil) via the CCS Manager software (Version 1.5.2.404; Stil). The setup has a resolution of 0.09 mN, a measurement range of 2550 mN in the friction direction, and a resolution of 0.05 mN and a range of 4800 mN in normal direction. The measurement frequency was 1000 Hz. The substrate (red in Figure 9) was mounted on a digitally controlled 2D translation stage (Thorlabs PT1/M-Z8, with additional KDC101 controllers, green in Figure 2), allowing for the controlled positioning of the substrate with respect to the sample mounted on the force transducer.

To assure proper alignment, the measuring platform (which had a size of 2 × 2 cm) was recorded with a Photron Fastcam SA-X2 camera (maximum resolution of 2000 × 2000 px), fitted with a Nikon Micro-Nikkor AF-S VR 105 mm *f*/2.8G lens and a 27.5 mm distance collar (Nikon PK-13), prior and during measuring, and real-time projected full-screen on a 22″ display.

**Figure 10:**
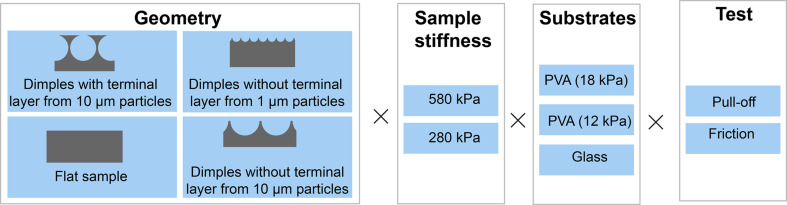
Overview of the tested conditions.

We measured pull-off and friction forces of the three types of micropatterns described above (1: dimples without a terminal layer from sub-microscale particles; 2: dimples with a terminal layer from microscale particles; and 3: dimples without a terminal layer from microscale particles) and of flat samples, fabricated from two crosslinker/pre-polymer weight ratios (1:10 and 1:20), on three substrates (PVA-12, PVA-18, and glass). An overview of the tested conditions is given in Figure 10.

Pull-off force was measured after preloading the sample with 55 mN for 10 s. The pull-off speed was 100 µm/s. Friction was measured using a load of 55 mN and a sliding speed of 500 µm/s. The peak pull-off and friction forces were derived from the recorded force curves.

The sample size was five. For each sample, both pull-off and friction forces were measured five consecutive times. Pull-off and friction forces were measured consecutively for each sample and in counterbalanced order across the samples. The conditions (4 geometries × 2 stiffness degrees of the sample × 3 substrates) were tested in randomized order. When measuring on PVA substrates, the substrate was left for 2 min between consecutive measurements to elastically recover. Humidity and temperature were kept constant during all measurements.

### Statistical analyses

All statistical analyses were conducted between samples, using the first of the five consecutively recorded peak (pull-off or friction) forces measured for each sample. We used the first of the five consecutively recorded peak forces instead of their mean, because a consistent decreasing trend was observed from the first to the fifth measurement (likely due to time-dependent stiffness and relaxation of the sample and the substrate), pointing towards a dependency between the consecutive measurements. Because the pull-off and friction measurement data may have unequal variances and/or be non-normally distributed, these data were rank-transformed (cf. Conover and Iman [47]) prior to being subjected to a three-way analysis of variance (ANOVA) with a post hoc Tukey–Kramer test to test the effects of geometry, sample stiffness, and substrate stiffness on pull-off and friction forces.

## Supporting Information

File 1 Additional experimental data.
