# Supplementary material for: Pull-off and friction forces of micropatterned elastomers on soft substrates: the effects of pattern length scale and stiffness
Source: Beilstein J Nanotechnol. 2019 Jan 8;10:79–94. doi: 10.3762/bjnano.10.8 (PMC6334791; doi:10.3762/bjnano.10.8)
Supplement: File 1 — Additional experimental data. [file Beilstein_J_Nanotechnol-10-79-s001.pdf]

BEILSTEIN JOURNAL OF NANOTECHNOLOGY

Supporting Information

for

Pull-off and friction forces of micropatterned elastomers on soft substrates: the effects of pattern length scale and stiffness

Peter van Assenbergh, Marike Fokker, Julian Langowski, Jan van Esch,
Marleen Kamperman and Dimitra Dodou

Beilstein J. Nanotechnol. **2019,** *10,* 79–94. doi:10.3762/bjnano.10.8

Additional experimental data

License and Terms: This is a supporting information file under the terms of the Creative Commons Attribution License (http://creativecommons.org/licenses/by/4.0). Please note that the reuse, redistribution and reproduction in particular requires that the authors and source are credited.
The license is subject to the *Beilstein Journal of Nanotechnology* terms and conditions: (https://www.beilstein-journals.org/bjnano)

1 Rheological characterization of poly(vinyl alcohol) (PVA) substrates (PVA-12 and PVA-18)

The storage and loss moduli of PVA subject to 2 and 3 freeze-thaw cycles were determined with a TA Instruments AR-G2 rheometer. A parallel plate geometry with diameter of 25 mm was used. Storage and loss moduli were determined at a strain of 0.05%, for a frequency range from $1{\cdot}10^{-1}$ to $1{\cdot}10^{2}$ rad/s.

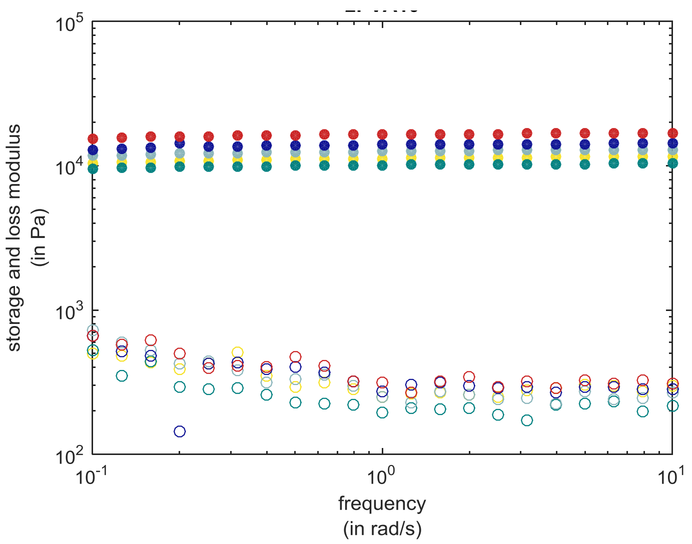

Figure S1: Storage modulus (•) and loss moduli (○) as a function of angular frequency for 5 samples of PVA samples subjected to 2 freeze-thaw cycles. The average storage modulus at 0.1 rad/s was 12.002 kPa.

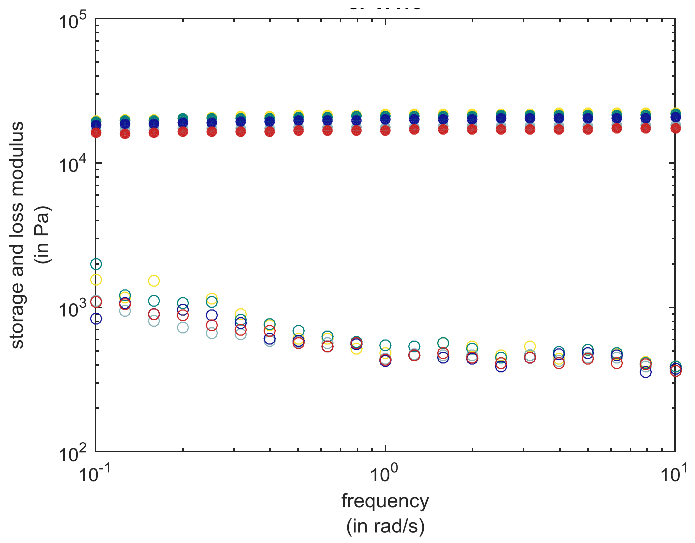

Figure S2: Storage modulus (•) and loss moduli (○) as a function of angular frequency for 5 samples of PVA samples subjected to 3 freeze-thaw cycles. The average storage modulus at 0.1 rad/s was 18.038 kPa.

Table S1: Average storage moduli for 5 PVA samples subjected to 2 freeze-thaw cycles, and 5 samples subjected to 3 freeze-thaw cycles.

	Mean (μ) at 0.1 rad/s [Pa]	Standard deviation
2 freeze-thaw cycles	12002	2.4661e+03
3 freeze-thaw cycles	18038	2.0692e+03

The mean storage moduli at 0.1 rad/s (750 μm/s) were compared. A two-sample t-test shows that the two means significantly differed ($t(8) = 4.61$; $p = 0.0012$).

Average Tan delta (G''/G') for PVA-12 at 0.1 rad/s was 0.0517.

Average Tan delta for PVA-18 at 0.1 rad/s was 0.0724.

2 Structural characterization of sub-micrometer dimple arrays with AFM

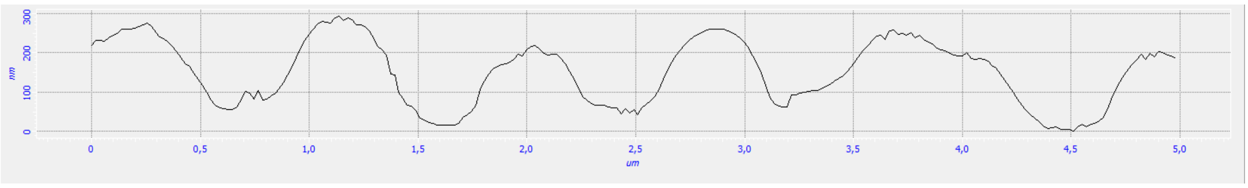

Figure S3: A cross section of a sub-micrometer dimple micropattern. The dimple depth is about 200 nm, and the center-to-center distance between dimples is about 0.8 µm.

3 The effect of curing at room temperature

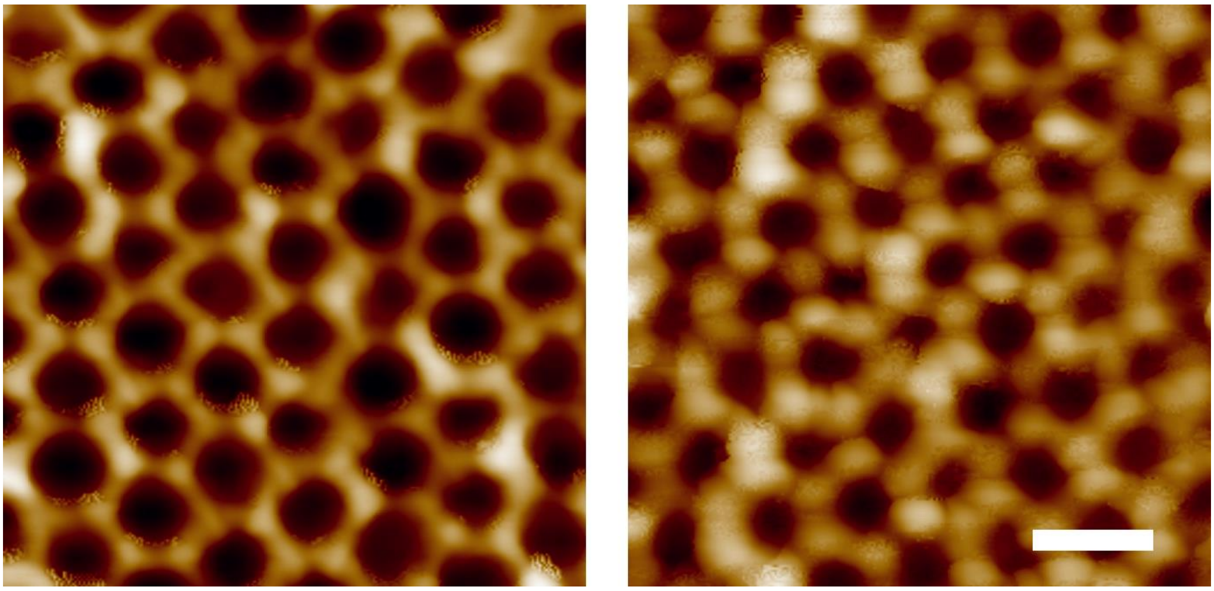

Figure S4: The effect of curing the elastomer at room temperature for 48 h (left) versus curing at 68 °C for 2 h (right). When curing at room temperature, the uncured elastomer remains in a liquid state much longer, allowing it to fully flow through the colloidal monolayer. This results in sharper edges separating neighboring dimples, and lager dimple diameters. Scale bars are 1 µm,

4 Sample fabrication and mounting

Fabrication of PDMS micropatterns

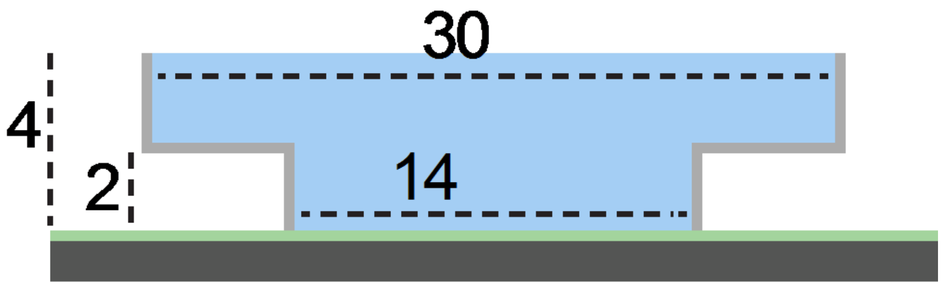

Figure S5: A two-layer squared frame (light grey) was placed on a glass slide (dark grey), coated with a colloidal monolayer (green). The frame was filled with uncured PDMS and the filled frame was placed in the oven for 2 h at 68.3 °C for curing. The dimensions of the mold are shown in millimeters.

Mounting of the sample on the force platform

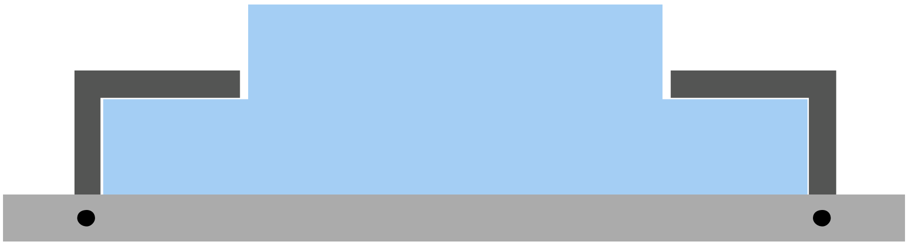

Figure S6: The fabricated PDMS sample (blue) was placed on the force platform (light grey). A covering part from stainless steel (SS) with a squared void (dark grey) was placed on top of the sample to fixate it. Small magnets (black) were integrated in the force platform to fixate the SS covering part on the force platform.

5 Additional Data

Pull-off data

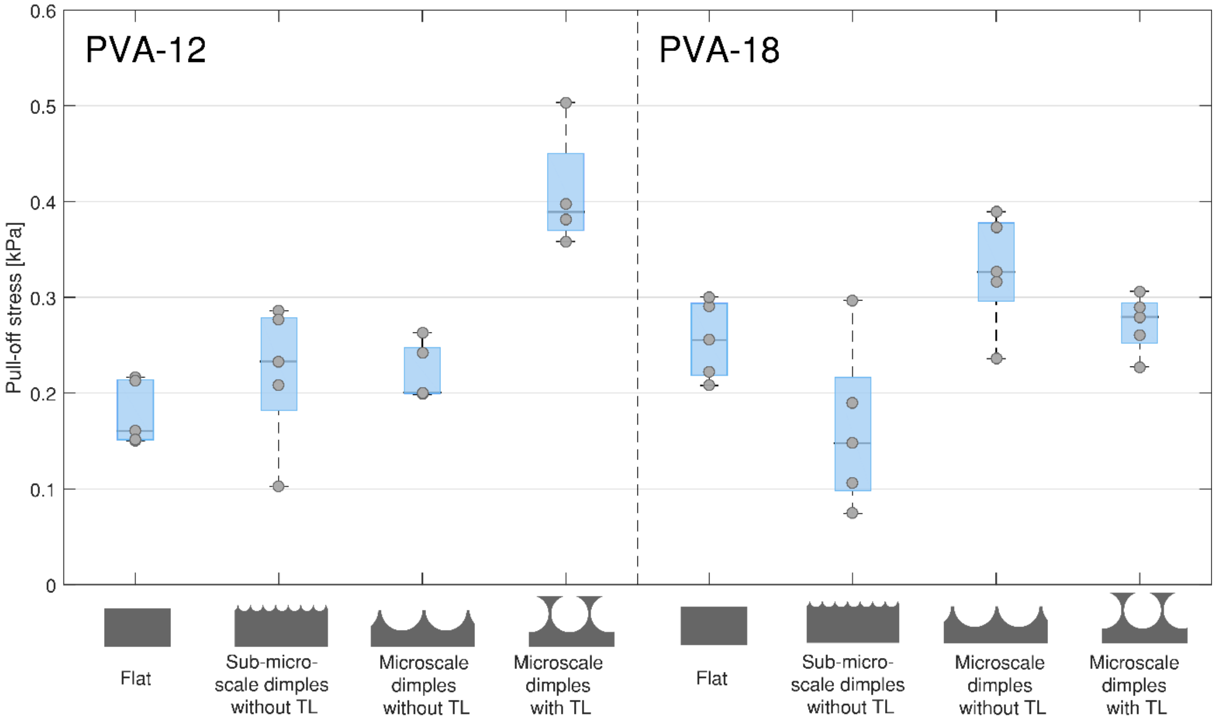

Figure S7: Pull-off stress for flat samples, sub-microscale dimples without terminal layer, and microscale dimples with and without terminal layer, all from PDMS-280 on PVA-12 (left) and PVA-18 (right). Each data point represents the average of five consecutive measurements of one sample, and in each boxplot, five different samples for each geometry are included.

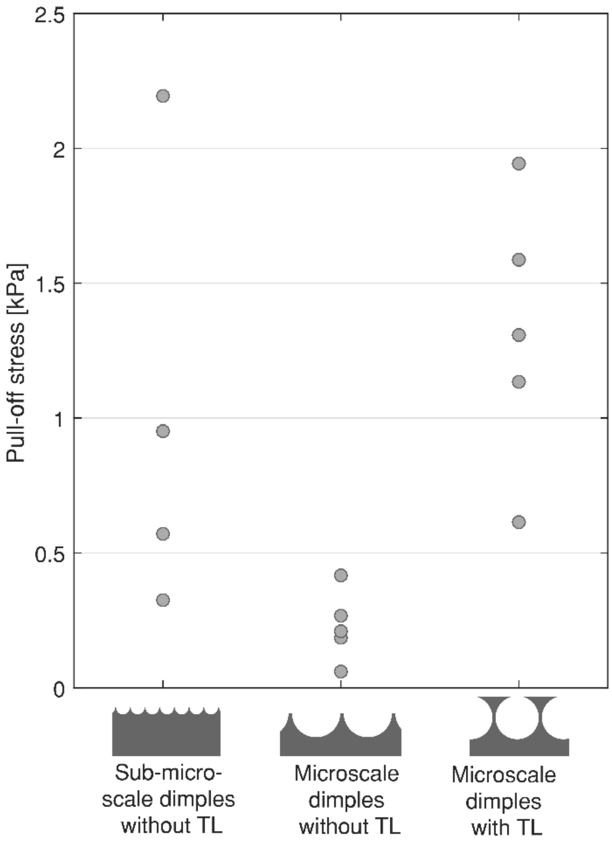

Figure S8: Pull-off stress for sub-microscale dimples without terminal layer, and microscale dimples with and without terminal layer, all from PDMS-280, on glass. Each data point represents the average of five consecutive measurements of one sample, and in each boxplot, five different samples for each geometry are included.

Table S2: Median and interquartile range (IQR) of pull-off forces (mN) of all fabricated micropatterns on two types of PVA.

	Substrate	PVA-12				PVA-18			
	**Sample stiffness**	**PDMS-280**		**PDMS-580**		**PDMS-280**		**PDMS-580**	
**Geometry**	**Size**	Median	IQR	Median	IQR	Median	IQR	Median	IQR
**Flat**	**-**	32.47	7.39	33.15	5.17	51.96*	16.42	57.75	9.05
**Dimples without TL**	**Sub-microscale**	45.83	19.88	45.04	26.89	28.97	21.27	56.90	16.87
**Dimples without TL**	**Microscale**	44.66	9.83	32.61**	7.36	68.19	20.31	60.83	16.35
**Dimples with TL**	**Microscale**	76.18*	17.13	63.66	13.42	57.76	15.26	44.58	21.72

TL: terminal layer; *one missing value; **two missing values.

Friction data

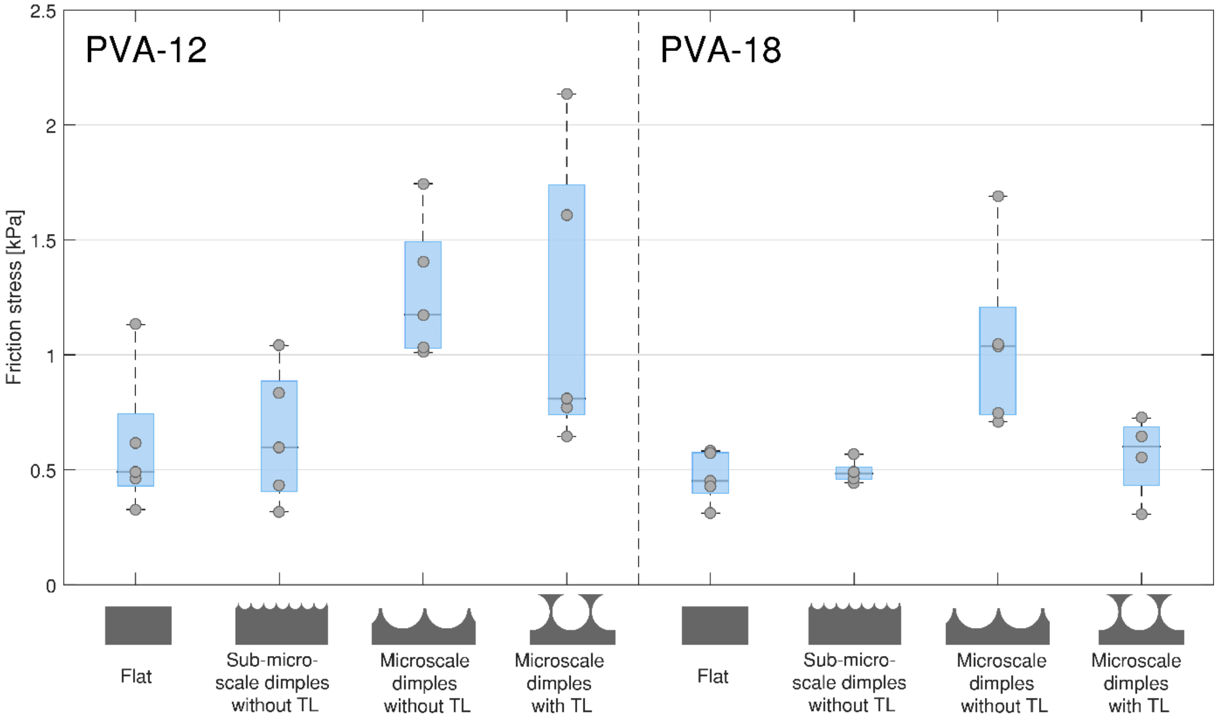

Figure S9: Friction stress for PDMS-280 micropatterns of sub-microscale dimples without terminal layer, and microscale dimples with and without terminal layer, on PVA-12 (left) and PVA-18 (right). Each data point represents the average of five consecutive measurements of one sample, and in each boxplot, five different samples for each geometry are included.

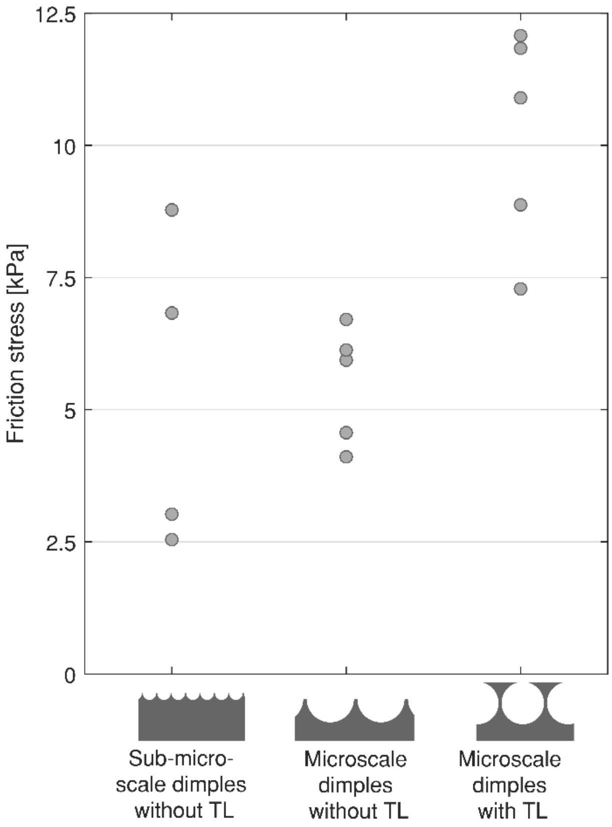

Figure S10: Friction stress for sub-microscale dimples without terminal layer, and microscale dimples with and without terminal layer, all from PDMS-280, on glass. Each data point represents the average of five consecutive measurements of one sample, and in each boxplot, five different samples for each geometry are included.

Table S3: Median and interquartile range (IQR) of friction forces (mN) of all fabricated micropatterns on two types of PVA.

	**Substrate**	**PVA-12**				**PVA-18**			
	**Sample stiffness**	**PDMS-280**		**PDMS-580**		**PDMS-280**		**PDMS-580**	
**Geometry**	**Size**	Median	IQR	Median	IQR	Median	IQR	Median	IQR
**Flat**	**-**	69.03	88.84	94.02	64.12	30.53	35.49	64.18	39.19
**Dimples without TL**	**Sub-microscale**	132.82	109.03	97.64	27.92	166.82	39.88	87.46	61.11
**Dimples without TL**	**Microscale**	254.06	100.02	183.00	55.18	135.84	43.79	249.16	98.75
**Dimples with TL**	**Microscale**	164.95	113.64	188.85	80.09	111.09	47.22	29.32	31.91

TL: terminal layer
